# A Recommended Numbering Scheme for Influenza A HA Subtypes

**DOI:** 10.1371/journal.pone.0112302

**Published:** 2014-11-12

**Authors:** David F. Burke, Derek J. Smith

**Affiliations:** Department of Zoology, University of Cambridge, Cambridge, United Kingdom; University of Edinburgh, United Kingdom

## Abstract

Comparisons of residues between sub-types of influenza virus is increasingly used to assess the zoonotic potential of a circulating strain and for comparative studies across subtypes. An analysis of N-terminal cleavage sites for thirteen subtypes of influenza A hemagglutinin (HA) sequences, has previously been described by Nobusawa and colleagues. We have expanded this analysis for the eighteen known subtypes of influenza. Due to differences in the length of HA, we have included strains from multiple clades of H1 and H5, as well as strains of H5 and H7 subtypes with both high and low pathogenicity. Analysis of known structures of influenza A HA enables us to define amino acids which are structurally and functionally equivalent across all HA subtypes using a numbering system based on the mature HA sequence. We provide a list of equivalences for amino acids which are known to affect the phenotype of the virus.

## Introduction

Increasingly, amino acid changes in HA, resulting from either natural evolution or experimental design, are compared to amino acids within another subtype. A common example are those mutations that have been shown to confer binding to human glycans. In strains from the H3 subtype, these are Gln226Leu and Gly228Ser whereas in strains from the H5 subtype these mutations are positions 222 and 224. Although simple 'rules-of-thumb' can be derived, such as the subtracting four from the H3 numbering to get the position in H5 viruses, this is not always straightforward, as typified by the recent focus on H7 viruses. The HA of H7 strains contain many amino acid insertions and deletions (indels) relative to viruses from the other subtypes. For amino acids close to the receptor binding site, such as the aforementioned mutations, the H7 numbering differs from H3 numbering by nine residues (Gln217 and Gly219). However, two other mutations of concern, His103Tyr and Thr315Ile, which were recently shown to facilitate the aerosol transmission of avian A/H5N1 viruses between mammals [1]–[2], lie in the N and C termini of HA1, respectively. Due to the indels in these regions, the equivalent amino acids in H7 strains differ by three (Gln100) and six (Thr309) amino acids, respectively. As shown for H7, the conversion of residue numbering between subtypes varies depending on the region of HA being compared. Yet another complication arises due to genetic changes within a subtype which, although uncommon, do occur. Over one-fifth of the avian H5N1 strains in the Middle East sequenced to date have a deletion between amino acids positions 128 and 130 (mature HA H5N1 numbering). This deletion was also found in human seasonal H1 strains after 1995 but was not present in early H1 strains or any of the H1pdm strains currently circulating [3]. Similarly, a clade of H7 strains circulating in North America and Canada since 1996 has been shown to have eight amino acids deleted, located surprisingly close to the receptor binding site [4]. Conversion rules thus also depend upon the lineage of the subtypes that are being compared.

Nobusawa and colleagues previously predicted the N-terminal sequence for thirteen subtypes of HA based on the likely signal peptide cleavage site of the N-terminal signal peptide [5], thus providing a numbering scheme based on the mature sequence of HA. Although widely cited, not all publications use this numbering. For example, only two (3M6S and 3ZTN) out of the thirteen currently available crystal structures of HA of the vaccine strain of H1pdm (A/California/04/2009) start with the mature HA sequence (Asp-Thr-Leu-Cys-Ile). Alternative structures include six (3AL4, 4JTV and 4JU0) or ten (3LZG, 3UBE, 3UBN, 3UBQ and 4F3Z) additional N-terminal amino acids. This variation in N-terminal numbering, in addition to subtype specific differences caused by indels, can increase confusion in interpreting amino acid equivalences. To avoid inaccuracies, it is important to have a scheme to define and compare numbering between subtypes.

Here we report an updated prediction of the proteolytic cleavage sites for all subtypes. We analyse known structures of HA to enable us to define amino acids which are structurally and functionally equivalent across the eighteen currently known subtypes of influenza A. Combining both of these results, we are able to compile a list of equivalences for amino acids which are known to affect the phenotype of the virus for all known HA subtypes.

## Materials and Methods

Representative sequences of HA for each subtype were downloaded from the Influenza Research Database (IRD). Potential N-terminal cleavage sites were predicted using the *signalP* [6]–[7] web-server. The amino acid sequence N-terminal to the predicted cleavage site was removed from each sequence. If a crystal structure was available, these were aligned based on their structural similarity using Pymol [8]. We then aligned the remaining sequences to the sequences of the other subtypes using FUGUE [9]. In general, amino acids in protein secondary structures (α-helices, β-strands) which are inaccessible to solvent or involved in interactions with other amino acids, are more conserved than those in loop regions or those exposed to solvent. Thus, amino acid insertions or deletions are more likely to occur solvent exposed regions or in regions without well-defined secondary structures. FUGUE uses knowledge of these differences in evolutionary constraints, in addition to sequence conservation, to aid its sequence alignment. This structure-based sequence alignment was subsequently manually adjusted based on inspection of the structures to accurately reflect structural similarity of loop regions.

## Results

We have re-analysed the predicted N-terminal signal peptide cleavage sites of subtypes H1 to H13 and have extended this analysis to include subtypes H14 to H18. Table 1 shows the signal peptide and N-terminal amino acid sequence of the mature protein based on the cleavage sites predicted using *signalP* [7]–[8], for each of the HA subtypes. More than half of all subtypes are predicted to be cleaved at an aspartic acid which is three amino acids N-terminal to a completely conserved cysteine. In agreement with Nobusawa, three subtypes are predicted to be cleaved at the amino acid preceding this aspartic acid at either a leucine (H10) or a tyrosine (H8 and H12). Three subtypes, H3, H5 and H14, lack the aspartic acid and are predicted to be cleaved at a glutamine, resulting in a longer mature N-terminal region. The signal peptide contains a stretch of about 10 hydrophobic amino acids that have a tendency to form a single alpha-helix, albeit with little sequence conservation between subtypes. In total, between 16 and 19 amino acids are removed from the N-terminal sequence to facilitate the movement of the virus through the ER membrane.

**Table 1:** Predicted signal peptide cleavage sites for all HA subtypes.

Subtype	Representative strain	Signal Peptide	N-terminal sequence of mature protein
H1	A/United Kingdom/1/1933	MKARLLVLLCALAATDA	DTICIGYHANNS
H2	A/Singapore/1/1957	MAIIYLILLFTAVRG	DQICIGYHANNS
H3	A/Aichi/2/1968	MKTIIALSYIFCLPLG	QDLPGNDNSTATLCLGHHAVPN
H4	A/swine/Ontario/01911–2/1999	MLSIAILFLLIAEGSS	QNYTGNPVICLGHHAVSN
H5	A/Vietnam/1203/2004	MEKIVLLFAIVSLVKS	DQICIGYHANNS
H6	A/chicken/Taiwan/0705/1999	MIAIIVIATLAAAGKS	DKICIGYHANNS
H7	A/Netherlands/219/2003	MNTQILVFALVASIPTNA	DKICLGHHAVSN
H8	A/turkey/Ontario/6118/1968	MEKFIAIAMLLASTNA	YDRICIGYQSNNS
H9	A/swine/Hong Kong/9/1998	MEAASLITILLVVTASNA	DKICIGYQSTNS
H10	A/mallard/bavaria/3/2006	MYKIVVIIALLGAVKG	LDKICLGHHAVAN
H11	A/duck/England/1/1956	MEKTLLFAAIFLCVKA	DEICIGYLSNNS
H12	A/duck/Alberta/60/1976	MEKFIILSTVLAASFA	YDKICIGYQTNNS
H13	A/gull/Maryland/704/1977	MALNVIATLTLISVCVHA	DRICVGYLSTNS
H14	A/mallard/Astrakhan/263/1982	MIALILVALALSHTAYS	QITNGTTGNPIICLGHHAVEN
H15	A/duck/Australia/341/1983	MNTQIIVILVLGLSMVRS	DKICLGHHAVAN
H16	A/black-headed-gull/Turkmenistan/13/1976	MMIKVLYFLIIVLGRYSKA	DKICIGYLSNNS
H17	A/little-yellow-shouldered bat/Guatemala/060/2010	MELIILLILLNPYTFVLG	DRICIGYQANQN
H18	A/flat-faced bat/Peru/033/2010	MITILILVLPIVVG	DQICIGYHSNNS

The N-terminal signal peptide cleavage site of HA was predicted using the *signalP* [7] for all HA subtypes. Most subtypes are cleaved close to a highly conserved aspartic acid. Three subtypes lacking this aspartic acid are cleaved at a glutamine resulting in a longer HA sequence.

**Figure 1:**
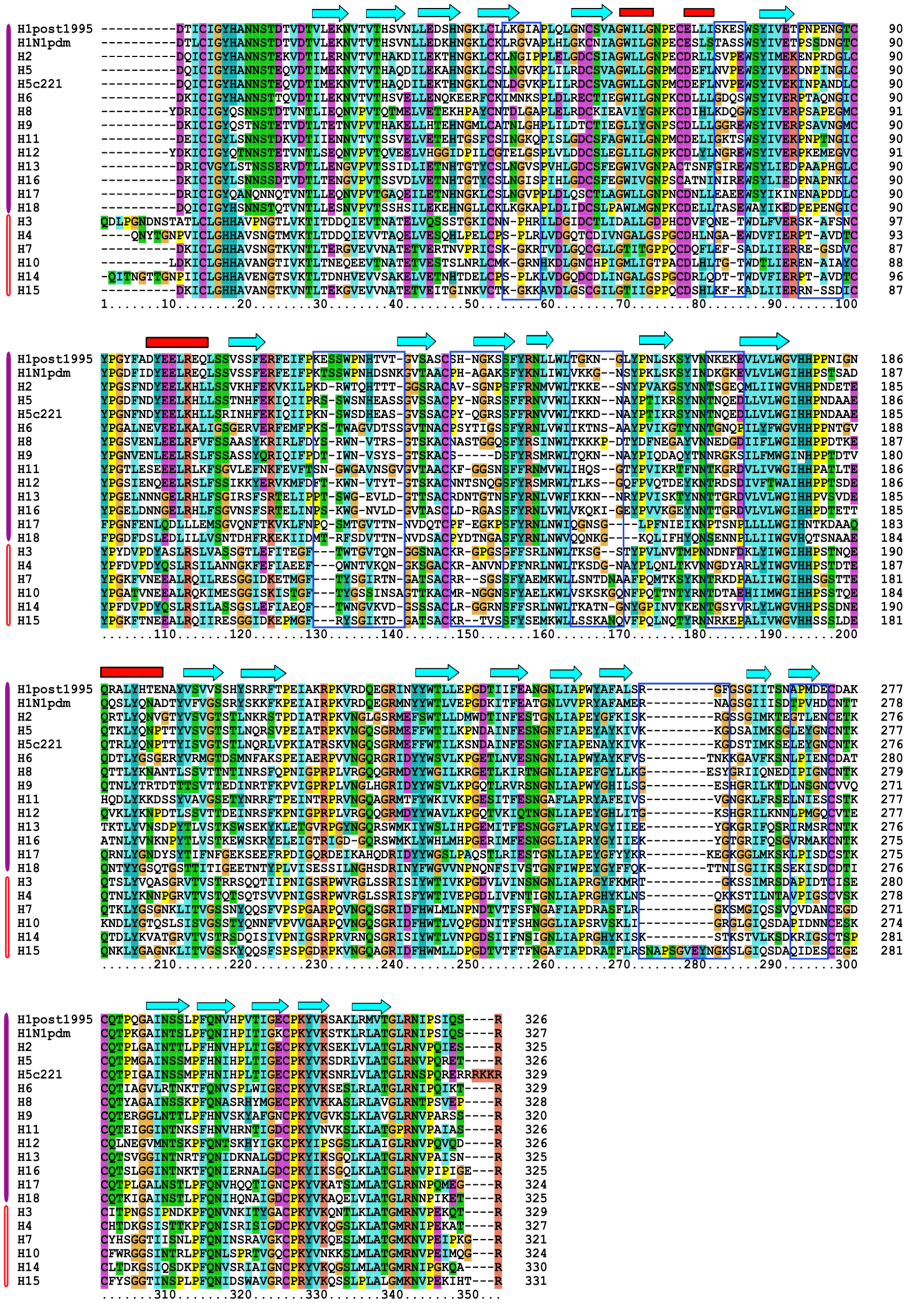
Sequence alignment of HA for known sub-types. Alignment of mature HA sequence for all known HA sub-types. Additional strains have been included for sub-types which show variation in the length of HA. Sequences are ordered according to their phylogenetic classification as group 1 (magenta bar) or group 2 (orange bar) HA. The protein secondary structure elements, α-helices and β-strands, are highlighted with red bars and cyan arrows, respectively. A blue box highlights regions which have high structural variation across all subtypes. Amino acids within these regions should not be defined as equivalent between all sub-types. Each amino acid is coloured according to clustalx2 rules [11]. Briefly, glycine and proline are coloured orange and yellow, respectively. Conserved positively charged residues and negatively charged residues are coloured red and magenta, respectively. Conserved cysteines are coloured pink while conserved serine or threonine residues are in green. The remaining amino acids, if conserved are coloured blue. The sequences representative of each subtype are as follows: H1(A/United Kingdom/1/1933); H1pdm(A/California/04/2009); H2(A/Singapore/1/1957); H3(A/Aichi/2/1968); H4(A/swine/Ontario/01911/2/1999); H5(A/Vietnam/1203/2004); H5c221(A/chicken/Egypt/0915-NLQP/2009); H6(A/chicken/Taiwan/0705/1999); H7(A/Netherlands/219/2003); H8(A/turkey/Ontario/6118/1968); H9(A/swine/HongKong/9/1998); H10(A/mallard/bavaria/3/2006); H11(A/duck/England/1/1956); H12(A/duck/Alberta/60/1976); H13(A/gull/Maryland/704/1977); H14(A/mallard/Astrakhan/263/1982); H15(A/duck/Australia/341/1983); H16(A/black-headed-gull/Turkmenistan/13/1976); H17(A/little-yellow-shouldered-bat/Guatemala/060/2010); H18(A/flat-facedbat/Peru/033/2010).

To define amino acids which are structurally equivalent across subtypes, we compared the available protein structures of all subtypes of HA to produce a sequence alignment based on the structural similarity of HA. For those subtypes without an HA structure (H4, H6, H8, H10–H18), we aligned their sequences to those of the other subtypes using an algorithm which considers structural features in addition to sequence conservation (see Material & Methods) [9]. The structure-based sequence alignment of HA1 is shown in figure 1. The subtypes have been ordered according to their phylogenetic grouping [10] and coloured according to sequence conservation [11]. We have highlighted those regions of HA which show significant differences in structure between strains of different subtypes. These are typically loops between secondary structures and are regions which contain insertions and deletions. Amino acids in these regions should only be considered to be equivalent when comparing closely related subtypes.

As previously described, some subtypes show clade specific differences in the length of the amino acid sequence of HA. We have therefore distinguished in our analysis H1 strains post-1995 and strains from clade 2.2.1 of H5. Additionally, the insertion of many positively charged amino acids in the C-terminal of HA1 in some strains of H5 and H7 subtypes is well known to increase the pathology of viral infection in poultry, leading to high rates of fatality [12]. A consequence is that the numbering of positions C-terminal to the cleavage site (position 326 for low pathogenic strains of H5) will differ. For H5 and H7 subtypes, we therefore also include both low-pathogenic (H5N1:A/mallard/Italy/3401/2005; H7:A/Turkey/Italy/220158/2002) and high-pathogenic (H5N1:A/Vietnam/1203/2004; H7N7:A/Netherlands/219/2003) strains. The sequence alignment including all subtypes spanning both HA1 and HA2 is available as File S1.

From these alignments, we can now derive residue numbering in each subtype, of every position of HA, relative to its mature sequence. This list of equivalences for all residue positions and across all subtypes are available as File S2 and at http://www.antigenic-cartography.org/surveillance/evergreen/HAnumbering. Positions which are most often compared across subtypes are those which have been shown to be associated with changes in phenotype. In 2012, the WHO Collaborating Center for Influenza Reference and Research at the Centers for Disease Control and Prevention in Atlanta compiled an inventory of amino acid mutations found in H5N1 viruses http://www.cdc.gov/flu/avianflu/h5n1/inventory.htm). The equivalent residue numbering for these mutations in HA are listed in Table 2 for those subtypes which circulate in humans (H1, H3) or from which zoonoses frequently occur (H5, H7, H9).

**Table 2:** Equivalent amino acid numbering for subtypes currently circulating in humans or have pandemic potential.

Mutation	H1pdm	H3	H5	H7	H9	Phenotype	Reference
Tyr → His	7	17	7	7	7	Increase in fusion pH	[13]
His → Gln	8	18	8	8	8	Decrease in fusion pH; increased stability	[13]
Asn → Any	11	21	11	11	11	Loss of N-glycosylation; increased virulence	[14]
Glu→Lys	75	83	75	73	75	Increased virus binding to α2-6 glycans	[15]
His → Tyr	103	110	103	100	103	Increased stability	[2]
Ser→ Asn	122	126	121	116	121	Increased virus binding to α2-6 glycans	[16]
Ser→Pro	124	128	123	118	123	Increased virus binding to α2-6 glycans	[15]
Ala → Δ	130	Δ	129	Δ	Δ	Increased virus binding to α2-6 glycans	[17]–[18]
Ser → Ala	134	137	133	127	131	Increased virus binding to α2-6 glycans	[19]
Ala→ Val	135	138	134	128	132	Increased infectivity in SIAT Cells	[20]
Gly→Arg	140	143	139	132	Δ	Increased virus binding to α2-6 glycans	[15]
Ile→ Thr	152	155	151	144	145	Increased virus binding to α2-6 glycans	[17]–[18]
Asn→ Asp	155	158	154	147	148	Loss of N-glycosylation; increased binding and transmission	[2]
Thr→ Ala	157	160	156	151	150	Loss of N-glycosylation; increased binding and transmission	[1]
Asn→Lys	183	186	182	177	176	Increased virus binding to α2-6 glycans	[15], [21]
Asp→Gly	184	187	183	178	177	Increased virus binding to α2-6 glycans	[22]
Glu→Gly	187	190	186	181	180	Increased virus binding to α2-6 glycans	[22]
Thr→Ile	189	192	188	183	182	Increased virus binding to α2-6 glycans	[19]
Lys→Arg	190	193	189	184	183	Increased virus binding to α2-6 glycans	[16]
Gln→Arg/His	193	196	192	187	186	Increased virus binding to α2-6 glycans	[15],
Asn→Lys	194	197	193	188	187	Increased virus binding to α2-6 glycans	[15]
Val → Ile	211	214	210	205	204	Increased virus binding to α2-6 glycans	[18]
Gln→Leu	223	226	222	217	216	Increased virus binding to α2-6 glycans	[21]
Ser→Asn	224	227	223	218	217	Increased virus binding to α2-6 glycans	[21]–[23]
Gly→Ser	225	228	224	219	218	Increased virus binding to α2-6 glycans	[14]–[15], [24]
Pro→Ser	236	239	235	230	229	Increased virus binding to α2-6 glycans	[18]
Glu→Lys	252	255	251	246	245	Increased virus binding to α2-6 glycans	[22]
Thr→Ile	316	318	315	309	309	Increase in fusion pH	[1]
Insertion of Arg or Lys	327	329	326	321	320	Poly-basic cleavage; increased pathogenicity	[25]
Lys → Ile	385	387	384	379	378	Increase in fusion pH; increased stability	[13], [26]
Asn → Lys	441	443	440	435	434	Increase in fusion pH; decreased stability	[13]
Asn → Asp	444	446	443	438	437	Increase in fusion pH	[27]
Arg → Lys	494	496	493	488	487	Increased virus binding to α2-6 glycans	[15]

Residue numbering is based on the mature sequence of HA1 across all subtypes for a set mutations shown to cause phenotypic differences. Positions where there is a deletion relative to other subtypes are represented by a “Δ”.

## Discussion

The length of the HA segment of influenza A shows substantial variation both between and within HA subtypes. This is caused by both changes in the length of the N-terminal signal peptide cleavage site and subtype specific amino acid insertions and deletions within the HA. These differences often makes it difficult to compare amino acid changes within HA of one subtype to those seen in another subtype.

We have re-assessed the predicted N-terminal signal peptide cleavage sites of all known subtypes (H1 to H18), confirming the previous definitions of the thirteen subtypes of HA previously reported by Nobusawa [5]. Using a structure-based approach we have analysed the structural and functional conservation of each position of HA across all subtypes. We have identified regions of HA which are structurally conserved across subtypes, including both low and highly pathogenic strains of H5 and H7 subtypes, and strains of H1 and H5 which show clade specific differences in the length of HA. From this data we have defined equivalent residue numbering for each subtype.

It is often stated that amino acid positions are 'equivalent' but rarely is this term defined explicitly. In structural biology, when comparing structures of proteins with evolutionary divergent sequences, such as HA from different subtypes, segments of the structure can be described as being either structurally conserved regions (SCRs) or structurally variable regions (SVRs). SCRs have similar structural features, such as the shape of the peptide backbone and the orientation of the sidechain atoms, and these regions usually have high sequence conservation. Like many proteins, the conserved regions within HA are those which are critical for its function, such as the receptor-binding site, or those that are required for the correct folding or stability of the protein structure. Amino acids within these regions can be described as equivalent in the sense that they will adopt nearly identical conformations and form similar interactions with other amino acids or bio-molecules. It is equally important to appreciate the limitations of a sequence alignment. Most alignment algorithms are parameterised to favour as few insertions and deletions as possible and do not always reflect local structural similarity. It is possible to have regions of sequences aligned which show little structural similarity and thus should not be described as SCRs. However, it needs to be noted that the SCR designation is not an absolute. Whilst many SCRs can be conserved across highly divergent sequences (between influenza A and influenza B viruses, for example), it is possible to define SCRs which are only conserved between closely related sequences, such as only between group 1 sub-types of HA.

In contrast, SVRs are regions which have very little structural or functional similarity between two related proteins. These regions are usually in the solvent exposed turns of the protein structure. These are also the regions where insertions and deletions of amino acids frequently occur, since they can be accommodated without major disruption of the fold or function of the protein. Amino acids in these regions should not be described as equivalent and comparisons between sub-types has little biological relevance.

Many studies attempt to compare, and sometimes replicate, mutations seen in one subtype, such as H5, to those in another subtype. Careful consideration of the level of structural and functional conservation of that region (its equivalence), however, is crucial. This is especially important when inferring analogous mutations from subtypes belonging to a different phylogenetic group. We feel that the use of this set of residue numbering and analysis of structural conservation will facilitate cross-subtype comparisons and reduce confusion in reporting amino acid numbering.

## Supporting Information Legends

File S1 **Structure based sequence alignment for HA.** The sequence alignment including all subtypes spanning both HA1 and HA2. This alignment includes a strain of seasonal H1N1 strain post-1995 (A/NewCaledonia/20/1999/H1N1) and strains of H5 (A/mallard/Italy/3401/2005/H5N1) and H7 (A/Turkey/Italy/220158/2002/H7N3) with low pathogenicity.
(DOC)

File S2 **Equivalent amino acid numbering for all known HA subtypes.** Residue numbering is based on the mature sequence of HA across all subtypes. The amino acid at each position for the representative strain of that subtype is also given. Positions where there is a deletion relative to other subtypes are represented by a “Δ”.
(XLS)
