# Supplementary material for: A Recommended Numbering Scheme for Influenza A HA Subtypes
Source: PLoS One. 2014 Nov 12;9(11):e112302. doi: 10.1371/journal.pone.0112302 (PMC4229193; doi:10.1371/journal.pone.0112302)
Supplement: File S1 — Structure based sequence alignment for HA. The sequence alignment including all subtypes spanning both HA1 and HA2. This alignment includes a strain of seasonal H1N1 strain post-1995 (A/NewCaledonia/20/1999/H1N1) and strains of H5 (A/mallard/Italy/3401/2005/H5N1) and H7 (A/Turkey/Italy/220158/2002/H7N3) with low pathogenicity. (DOC) [file pone.0112302.s001.doc]

>A/Swine/Iowa/15/1930/H1N1

----------DTLCIGYHANNSTDTVDTVLEKNVTVTHSVNLLEDSHNGKLCRLGGIAPLQLGKCNIAGXXLGNPECDLLLTVSSWSYIVETSNSDNGTCYPGDFIDYEELREQLSSVSSFEKFEIFPKTSSWPNHETTRGVTAACPY-AGASSFYRNLLWLVKKE--NSYPKLSKSYVNNKGKEVLVLWGVHHPPTSTDQQSLYQNADAYVSVGSSKYDRRFTPEIAARPKVRGQAGRMNYYWTLLEPGDTITFEATGNLVAPRYAFALNR---------GSESGIITSDAPVHDCDTKCQTPHGAINSSLPFQNIHPVTIGECPKYVKSTKLRMVTGLRNIPSIQS----RGLFGAIAGFIEGGWTGLIDGWYGYHHQNGQGSGYAADQKSTQNAIDGITNKVNSVIEKMNTQFTVVGKEFNNLERRIKNLNKKVDDGFLDVWTYNAEMLVLLENERTLDFHDSNVKNLYEKARSQLRNNAKEIGNGCFEFYHKCDDACMESVRNGTYDYPKYSEESKLNREEIDGVKLESMM-VYQILAIYSTVASSLVLLVSLGAISFWMCSNGSLQCRICI

>A/Puerto/Rico/8/1934/H1N1

----------DTICIGYHANNSTDTVDTVLEKNVTVTHSVNLLEDSHNGKLCRLKGIAPLQLGKCNIAGWLLGNPECDPLLPVRSWSYIVETPNSENGICYPGDFIDYEELREQLSSVSSFERFEIFPKESSWPNHNTNRGVTAACSH-EGKSSFYRNLLWLTEKE--GSYPKLKNSYVNKKGKEVLVLWGIHHPPNSKEQQNLYQNENAYVSVVTSNYNRRFTPEIAERPKVRDQAGRMNYYWTLLKPGDTIIFEANGNLIAPMYAFALRR---------GFGSGIITSNASMHECNTKCQTPLGAINSSLPYQNIHPVTIGECPKYVRSAKLRMVTGLRNIPSIQS----RGLFGAIAGFIEGGWTGMIDGWYGYHHQNEQGSGYAADQKSTQNAINGITNKVNSVIEKMNIQFTAVGKEFNKLEKRMENLNNKVDDGFLDIWTYNAELLVLLENERTLDFHDSNVKNLYEKVKSQLKNNAKEIGNGCFEFYHKCDNECMESVRNGTYDYPKYSEESKLNREKVDGVKLESMG-IYQILAIYSTVASSLVLLVSLGAISFWMCSNGSLQCRICI

>A/United/Kingdom/1/1933/H1N1

----------DTICIGYHANNSTDTVDTLLEKNVTVTHSVNLLEDSHNGKLCKLKGIAPLQLGKCNIAGWLLGNPECDSLLPARSWSYIVETPNSENGACYPGDFIDYEELREQLSTVSSLERFEIFPKESSWPNHNTLKGVTVSCSH-GGKSSFYRNLLWLTKTG--DSYPKLNNSYVNNKGKEVLVLWGVHHPSSSNEQQSLYHNVNAYVSVVSSNYNRRFTPEIAARPKVRDQPGRMNYYWTLLEPGDTILFEATGNLIAPWYAFALSR---------GFGSGIITSNASMHECNTKCQTPQGAINSSLPFQNIHPVTIGECPKYVRSTKLRMVTGLRNIPSIQS----RGLFGAIAGFIEGGWTGMIDGWYGYHHQNEQGSGYAADQKSTQNAINGITNKVNSVIEKMNTQFTAVGKEFNNLEKRMENLNKKVDDGFLDIWTYNAELLVLLENERTLDFHDLNVKNLYEKVKSQLKNNAKEIGNGCFEFYHKCDNECMESVRDGTYDYPKYSEESKLNREKIDGVKLESMG-VYQILAIYSTVASSLVLLVSLGAISFWMCSNGSLQCRICI

>A/NewCaledonia/20/1999/H1N1

----------DTICIGYHANNSTDTVDTVLEKNVTVTHSVNLLEDSHNGKLCLLKGIAPLQLGNCSVAGWILGNPECELLISKESWSYIVETPNPENGTCYPGYFADYEELREQLSSVSSFERFEIFPKESSWPNHTVT-GVSASCSH-NGKSSFYRNLLWLTGKN--GLYPNLSKSYVNNKEKEVLVLWGVHHPPNIGNQRALYHTENAYVSVVSSHYSRRFTPEIAKRPKVRDQEGRINYYWTLLEPGDTIIFEANGNLIAPWYAFALSR---------GFGSGIITSNAPMDECDAKCQTPQGAINSSLPFQNVHPVTIGECPKYVRSAKLRMVTGLRNIPSIQS----RGLFGAIAGFIEGGWTGMVDGWYGYHHQNEQGSGYAADQKSTQNAINGITNKVNSVIEKMNTQFTAVGKEFNKLERRMENLNKKVDDGFLDIWTYNAELLVLLENERTLDFHDSNVKNLYEKVKSQLKNNAKEIGNGCFEFYHKCNNECMESVKNGTYDYPKYSEESKLNREKIDGVKLESMG-VYQILAIYSTVASSLVLLVSLGAISFWMCSNGSLQCRICI

>A/California/04/2009/H1N1pdm

----------DTLCIGYHANNSTDTVDTVLEKNVTVTHSVNLLEDKHNGKLCKLRGVAPLHLGKCNIAGWILGNPECESLSTASSWSYIVETPSSDNGTCYPGDFIDYEELREQLSSVSSFERFEIFPKTSSWPNHDSNKGVTAACPH-AGAKSFYKNLIWLVKKG--NSYPKLSKSYINDKGKEVLVLWGIHHPSTSADQQSLYQNADTYVFVGSSRYSKKFKPEIAIRPKVRDQEGRMNYYWTLVEPGDKITFEATGNLVVPRYAFAMER---------NAGSGIIISDTPVHDCNTTCQTPKGAINTSLPFQNIHPITIGKCPKYVKSTKLRLATGLRNIPSIQS----RGLFGAIAGFIEGGWTGMVDGWYGYHHQNEQGSGYAADLKSTQNAIDEITNKVNSVIEKMNTQFTAVGKEFNHLEKRIENLNKKVDDGFLDIWTYNAELLVLLENERTLDYHDSNVKNLYEKVRSQLKNNAKEIGNGCFEFYHKCDNTCMESVKNGTYDYPKYSEEAKLNREEIDGVKLESTR-IYQILAIYSTVASSLVLVVSLGAISFWMCSNGSLQCRICI

>A/Singapore/1/1957/H2N2

----------DQICIGYHANNSTEKVDTILERNVTVTHAKDILEKTHNGKLCKLNGIPPLELGDCSIAGWLLGNPECDRLLSVPEWSYIMEKENPRDGLCYPGSFNDYEELKHLLSSVKHFEKVKILPKD-RWTQHTTT-GGSRACAV-SGNPSFFRNMVWLTKKE--SNYPVAKGSYNNTSGEQMLIIWGVHHPNDETEQRTLYQNVGTYVSVGTSTLNKRSTPDIATRPKVNGLGSRMEFSWTLLDMWDTINFESTGNLIAPEYGFKISK---------RGSSGIMKTEGTLENCETKCQTPLGAINTTLPFHNVHPLTIGECPKYVKSEKLVLATGLRNVPQIES----RGLFGAIAGFIEGGWQGMVDGWYGYHHSNDQGSGYAADKESTQKAFDGITNKVNSVIEKMNTQFEAVGKEFSNLERRLENLNKKMEDGFLDVWTYNAELLVLMENERTLDFHDSNVKNLYDKVRMQLRDNVKELGNGCFEFYHKCDDECMNSVKNGTYDYPKYEEESKLNRNEIKGVKLSSMG-VYQILAIYATVAGSLSLAIMMAGISFWMCSNGSLQCRICI

>A/mallard/Italy/3401/2005/H5N1

----------DQICIGYHANNSTEQVDTIMEKNVTVTHAQDILEKAHNGKLCSLNGVKPLILRDCSVAGWLLGNPMCDEFLNVPEWSYIVEKDNPINGLCYPGDFNDYEELKHLLSSTNHFEKIQIIPRS-SWSNHEASSGVSSACPY-NGRSSFFRNVVWLIKKN--NAYPTIKRSYNNTNQEDLLVLWGIHHPNDAAEQTKLYQNPTTYVSVGTSTLNQRSVPEIATRPKVNGQSGRMEFFWTILKPNDAINFESNGNFIAPEYAYKIVK---------KGDSAIMKSGLEYGNCNTKCQTPMGAINSSMPFHNIHPLTIGECPKYVKSDRLVLATGLRNVPQRET----RGLFGAIAGFIEGGWQGMVDGWYGYHHSNEQGSGYAADKESTQKAIDGITNKVNSITDKMNTQFEAVGKEFNNLERRIENLNKKMEDGFLDVWTYNAELLVLMENERTLDFHDSNVKNLYDKVRLQLRDNAKELGNGCFEFYHKCDNECMESVRNGTYDYPQYSEEARLNREEISGVKLESMG-TYQILSIYSTVASSLALAIMIAGLSFWMCSNGSLQCRICI

>A/Vietnam/1203/2004/H5N1

----------DQICIGYHANNSTEQVDTIMEKNVTVTHAQDILEKKHNGKLCDLDGVKPLILRDCSVAGWLLGNPMCDEFINVPEWSYIVEKANPVNDLCYPGDFNDYEELKHLLSRINHFEKIQIIPKS-SWSSHEASLGVSSACPY-QGKSSFFRNVVWLIKKN--STYPTIKRSYNNTNQEDLLVLWGIHHPNDAAEQTKLYQNPTTYISVGTSTLNQRLVPRIATRSKVNGQSGRMEFFWTILKPNDAINFESNGNFIAPEYAYKIVK---------KGDSTIMKSELEYGNCNTKCQTPMGAINSSMPFHNIHPLTIGECPKYVKSNRLVLATGLRNSPQRERRRKKRGLFGAIAGFIEGGWQGMVDGWYGYHHSNEQGSGYAADKESTQKAIDGVTNKVNSIIDKMNTQFEAVGREFNNLERRIENLNKKMEDGFLDVWTYNAELLVLMENERTLDFHDSNVKNLYDKVRLQLRDNAKELGNGCFEFYHKCDNECMESVRNGTYDYPQYSEEARLKREEISGVKLESIG-IYQILSIYSTVASSLALAIMVAGLSLWMCSNGSLQCRICI

>A/chicken/Egypt/0915-NLQP/2009/H5N1

----------DQICIGYHANNSTEQVDTIMEKNVTVTHAQDILEKTHNGKLCNLDGVKPLILRDCSVAGWLLGNPMCDEFLNVPEWSYIVEKINPANDLCYPGNFNDYEELKHLLSRINHFEKIQIIPKN-SWSDHEAS-GVSSACPY-QGRSSFFRNVVWLTKKD--NAYPTIKRSYNNTNQEDLLVLWGIHHPNDAAEQTRLYQNPTTYISVGTSTLNQRLVPKIATRSKVNGQSGRMEFFWTILKSNDAINFESNGNFIAPENAYKIVK---------KGDSTIMKSELEYGNCNTKCQTPIGAINSSMPFHNIHPLTIGECPKYVKSNRLVLATGLRNSPQRERRRKKRGLFGAIAGFIEGGWQGMVDGWYGYHHSNEQGSGYAADKESTQKAIDGVTNKVNSIIDKMNTQFEAVGREFNNLERRIENLNKKMEDGFLDVWTYNAELLVLMENERTLDFHDSNVKNLYDKVRLQLRDNAKELGNGCFEFYHRCDNECMESVRNGTYDYPQYSEEARLKREEISGVKLESIG-TYQILSIYSTVASSLALAIMVAGLFLWMCSNGSLQCRI--

>A/chicken/Taiwan/0705/1999/H6N1

----------DKICIGYHANNSTTQVDTILEKNVTVTHSVELLENQKEERFCKIMNKSPLDLRECTIEGWILGNPKCDLLLGDQSWSYIVERPTAQNGICYPGALNEVEELKALIGSGERVERFEMFPKS-TWAGVDTSSGVTNACPSYTIGSSFYRNLVWIIKTNS-AAYPVIKGTYNNTGNQPILYFWGVHHPPNTGVQDTLYGSGERYVRMGTDSMNFAKSPEIAERPVVNGQRGRIDYYWSVLKPGETLNVESNGNLIAPWYAYKFVS--------TNKKGAVFKSNLPIENCDATCQTIAGVLRTNKTFQNVSPLWIGECPKYVKSESLRLATGLRNIPQIKT----RGIFGAIAGFIEGGWTGMIDGWYGYHHENSQGSGYAADRESTQKAIDGITNKVNSIIDKMNTQFEAVDHEFSNLERRIGNLNKRMEDGFLDVWTYNAELLVLLENERTLDLHDANVKNLYEKVKSQLRDNANDLGNGCFEFWHKCDNECIESVKNGTYDYPKYQDESKLNRHKIESVKLENLG-VYQILAIYSTVSSSLVLVGLIIAIGLWMCSNGSMQCRICI

>A/turkey/Ontario/6118/1968/H8N4

---------YDRICIGYQSNNSTDTVNTLIEQNVPVTQTMELVETEKHPAYCNTDLGAPLELRDCKIEAVIYGNPKCDIHLKDQGWSYIVERPSAPEGMCYPGSVENLEELRFVFSSAASYKRIRLFDYS-RWN-VTRS-GTSKACNASTGGQSFYRSINWLTKKKP-DTYDFNEGAYVNNEDGDIIFLWGIHHPPDTKEQTTLYKNANTLSSVTTNTINRSFQPNIGPRPLVRGQQGRMDYYWGILKRGETLKIRTNGNLIAPEFGYLLKG--------ESYGRIIQNEDIPIGNCNTKCQTYAGAINSSKPFQNASRHYMGECPKYVKKASLRLAVGLRNTPSVEP----RGLFGAIAGFIEGGWSGMIDGWYGFHHSNSEGTGMAADQKSTQEAIDKITNKVNNIVDKMNREFEVVNHEFSEVEKRINMINDKIDDQIEDLWAYNAELLVLLENQKTLDEHDSNVKNLFDEVKRRLSANAIDAGNGCFDILHKCDNECMETIKNGTYDHKEYEEEAKLERSKINGVKLEENT-TYKILSIYSTVAASLCLAILIAGGLILGMQNGSCRCMFCI

>A/Swine/HK/9/1998/H9N2

----------DKICIGYQSTNSTETVDTLTETNVPVTHAKELLHTEHNGMLCATNLGHPLILDTCTIEGLIYGNPSCDLLLGGREWSYIVERPSAVNGMCYPGNVENLEELRSLFSSASSYQRIQIFPDT-IWN-VSYS-GTSKACS-----DSFYRSMRWLTQKN--NAYPIQDAQYTNNRGKSILFMWGINHPPTDTVQTNLYTRTDTTTSVTTEDINRTFKPVIGPRPLVNGLHGRIDYYWSVLKPGQTLRVRSNGNLIAPWYGHILSG---------ESHGRILKTDLNSGNCVVQCQTERGGLNTTLPFHNVSKYAFGNCPKYVGVKSLKLAVGLRNVPARSS----RGLFGAIAGFIEGGWPGLVAGWYGFQHSNDQGVGMAADRDSTQKAIDKITSKVNNIVDKMNKQYGIIDHEFSEIETRLNMINNKIDDQIQDIWTYNAELLVLLENQKTLDEHDANVNNLYNKVKRALGSNAMEDGKGCFELYHKCDDQCMETIRNGTYNRRKYKEESKLERQKIEGIKLESEG-TYKILTIYSTVASSLVIAMGFAAFLFWAMS----------

>A/duck/England/1/1956/H11N6

----------DEICIGYLSNNSTDKVDTIIENNVTVTSSVELVETEHTGSFCSINGKQPISLGDCSFAGWILGNPMCDELIGKTSWSYIVEKPNPTNGICYPGTLESEEELRLKFSGVLEFNKFEVFTSN-GWGAVNSGVGVTAACKF-GGSNSFFRNMVWLIHQS--GTYPVIKRTFNNTKGRDVLIVWGIHHPATLTEHQDLYKKDSSYVAVGSETYNRRFTPEINTRPRVNGQAGRMTFYWKIVKPGESITFESNGAFLAPRYAFEIVS---------VGNGKLFRSELNIESCSTKCQTEIGGINTNKSFHNVHRNTIGDCPKYVNVKSLKLATGPRNVPAIAS----RGLFGAIAGFIEGGWPGLINGWYGFQHRNEEGTGIAADKESTQKAIDQITSKVNNIVDRMNTNFESVQHEFSEIEERINQLSKHVDDSVVDIWSYNAQLLVLLENEKTLDLHDSNVRNLHEKVRRMLKDNAKDEGNGCFTFYHKCDNKCIERVRNGTYDHKEFEEESKINRQEIEGVKLDSSGNVYKILSIYSCIASSLVLAALIMGFMFWACSNGSCRCTICI

>A/Duck/Alberta/60/1976/H12N5

---------YDKICIGYQTNNSTETVNTLSEQNVPVTQVEELVHGGIDPILCGTELGSPLVLDDCSLEGLILGNPKCDLYLNGREWSYIVERPKEMEGVCYPGSIENQEELRSLFSSIKKYERVKMFDFT-KWN-VTYT-GTSKACNNTSNQGSFYRSMRWLTLKS--GQFPVQTDEYKNTRDSDIVFTWAIHHPPTSDEQVKLYKNPDTLSSVTTDEINRSFKPNIGPRPLVRGQQGRMDYYWAVLKPGQTVKIQTNGNLIAPEYGHLITG---------KSHGRILKNNLPMGQCVTECQLNEGVMNTSKPFQNTSKHYIGKCPKYIPSGSLKLAIGLRNVPQVQD----RGLFGAIAGFIEGGWPGLVAGWYGFQHQNAEGTGIAADRDSTQRAIDNMQNKLNNVIDKMNKQFEVVNHEFSEVESRINMINSKIDDQITDIWAYNAELLVLLENQKTLDEHDANVRNLHDRVRRVLRENAIDTGDGCFEILHKCDNNCMDTIRNGTYNHKEYEEESKIERQKVNGVKLEENS-TYKILSIYSSVASSLVLLLMIIGGFIFGCQNGNVRCTFCI

>A/gull/Maryland/704/1977/H13N6

----------DRICVGYLSTNSSERVDTLLENGVPVTSSIDLIETNHTGTYCSLNGVSPVHLGDCSFEGWIVGNPACTSNFGIREWSYLIEDPAAPHGLCYPGELNNNGELRHLFSGIRSFSRTELIPPT-SWG-EVLD-GTTSACRDNTGTNSFYRNLVWFIKKN--NRYPVISKTYNNTTGRDVLVLWGIHHPVSVDETKTLYVNSDPYTLVSTKSWSEKYKLETGVRPGYNGQRSWMKIYWSLIHPGEMITFESNGGFLAPRYGYIIEE---------YGKGRIFQSRIRMSRCNTKCQTSVGGINTNRTFQNIDKNALGDCPKYIKSGQLKLATGLRNVPAISN----RGFFGAIAGFIEGGWPGLINGWYGFQHQNEQGTGIAADKESTQKAIDQITTKINNIIDKMNGNYDSIRGEFNQVEKRINMLADRIDDAVTDIWSYNAKLLVLLENDKTLDMHDANVKNLHEQVRRELKDNAIDEGNGCFELLHKCNDSCMETIRNGTYDHTEYAEESKLKRQEIDGIKLKSEDNVYKALSIYSCIASSVVLVGLILSFIMWACSSGNCRFNVCI

>A/black-headedgull/Turkmenistan/13/1976/H16N3

----------DKICIGYLSNNSSDTVDTLTENGVPVTSSVDLVETNHTGTYCSLNGISPIHLGDCSFEGWIVGNPSCATNINIREWSYLIEDPNAPNKLCYPGELDNNGELRHLFSGVNSFSRTELINPS-KWG-NVLD-GVTASCLD-RGASSFYRNLVWLVKQKI-GEYPVVKGEYNNTTGRDVLVLWGIHHPDTETTATNLYVNKNPYTLVSTKEWSKRYELEIGTRIGD-GQRSWMKLYWHLMHPGERIMFESNGGLIAPRYGYIIEK---------YGTGRIFQSGVRMAKCNTKCQTSLGGINTNKTFQNIERNALGDCPKYIKSGQLKLATGLRNVPIPIGE---RGLFGAIAGFIEGGWPGLINGWYGFQHQNEQGTGIAADKASTQKAINEITTKINNIIEKMNGNYDSIRGEFNQVEKRINMLADRVDDAVTDIWSYNAKLLVLIENDRTLDLHDANVRNLHDQVKRALKSNAIDEGDGCFNLLHKCNDSCMETIRNGTYNHEDYREESQLKRQEIEGIKLKTEDNVYKVLSIYSCIASSIVLVGLILAFIMWACSNGSCRFNVCI

>A/little-yellow-shoulderedbat/Guatemala/060/2010/H17

----------DRICIGYQANQNNQTVNTLLEQNVPVTGAQEILETNHNGKLCSLNGVPPLDLQSCTLAGWLLGNPNCDNLLEAEEWSYIKINENAPDDLCFPGNFENLQDLLLEMSGVQNFTKVKLFNPQ-SMTGVTTN-NVDQTCPF-EGKPSFYRNLNWIQGNSG----LPFNIEIKNPTSNPLLLLWGIHNTKDAAQQRNLYGNDYSYTIFNFGEKSEEFRPDIGQRDEIKAHQDRIDYYWGSLPAQSTLRIESTGNLIAPEYGFYYKR--------KEGKGGLMKSKLPISDCSTKCQTPLGALNSTLPFQNVHQQTIGNCPKYVKATSLMLATGLRNNPQMEG----RGLFGAIAGFIEGGWQGMIDGWYGYHHENQEGSGYAADKEATQKAVDAITNKVNSIIDKMNSQFESNIKEFNRLELRIQHLSDRVDDALLDIWSYNTELLVLLENERTLDFHDANVKNLFEKVKAQLKDNAIDEGNGCFLLLHKCNNSCMDDIKNGTYKYMDYREESHIEKQKIDGVKLTDYS-RYYTMTLYSTIASSVVLGSLIIAAFLWGCQKGSIQCKICI

>A/flat-faced-bat/Peru/033/2010/H18

----------DQICIGYHSNNSTQTVNTLLESNVPVTSSHSILEKEHNGLLCKLKGKAPLDLIDCSLPAWLMGNPKCDELLTASEWAYIKEDPEPENGICFPGDFDSLEDLILLVSNTDHFRKEKIIDMT-RFSDVTTN-NVDSACPYDTNGASFYRNLNWVQQNKG----KQLIFHYQNSENNPLLIIWGVHQTSNAAEQNTYYGSQTGSTTITIGEETNTYPLVISESSILNGHSDRINYFWGVVNPNQNFSIVSTGNFIWPEYGYFFQK--------TTNISGIIKSSEKISDCDTICQTKIGAINSTLPFQNIHQNAIGDCPKYVKAQELVLATGLRNNPIKET----RGLFGAIAGFIEGGWQGLIDGWYGYHHQNSEGSGYAADKEATQKAVDAITTKVNNIIDKMNTQFESTAKEFNKIEMRIKHLSDRVDDGFLDVWSYNAELLVLLENERTLDFHDANVNNLYQKVKVQLKDNAIDMGNGCFKILHKCNNTCMDDIKNGTYNYYEYRKESHLEKQKID-------------------------------------------------

>A/AICHI/2/1968/H3N2

QDLPGNDNSTATLCLGHHAVPNGTLVKTITDDQIEVTNATELVQSSSTGKICNN-PHRILDGIDCTLIDALLGDPHCDVFQNE-TWDLFVERSK-AFSNCYPYDVPDYASLRSLVASSGTLEFITEGF---TWTGVTQN-GGSNACKR-GPGSGFFSRLNWLTKSG--STYPVLNVTMPNNDNFDKLYIWGIHHPSTNQEQTSLYVQASGRVTVSTRRSQQTIIPNIGSRPWVRGLSSRISIYWTIVKPGDVLVINSNGNLIAPRGYFKMRT----------GKSSIMRSDAPIDTCISECITPNGSIPNDKPFQNVNKITYGACPKYVKQNTLKLATGMRNVPEKQT----RGLFGAIAGFIENGWEGMIDGWYGFRHQNSEGTGQAADLKSTQAAIDQINGKLNRVIEKTNEKFHQIEKEFSEVEGRIQDLEKYVEDTKIDLWSYNAELLVALENQHTIDLTDSEMNKLFEKTRRQLRENAEEMGNGCFKIYHKCDNACIESIRNGTYDHDVYRDEALNNRFQIKGVELKSG--YKDWILWISFAISCFLLCVVLLGFIMWACQRGNIRCNICI

>A/swine/Ontario/01911-1/1099/H4N6

----QNYTGNPVICLGHHAVSNGTMVKTLTDDQIEVVTAQELVESQHLPELCPS-PLRLVDGQTCDIVNGALGSPGCDHLNGA-EWDVFIERPT-AVDTCYPFDVPDYQSLRSILANNGKFEFIAEEF---QWNTVKQN-GKSGACKR-ANVNDFFNRLNWLTKSDG-NAYPLQNLTKVNNGDYARLYIWGVHHPSTDTEQTNLYKNNPGRVTVSTQTSQTSVVPNIGSRPWVRGLSSRISFYWTIVEPGDLIVFNTIGNLIAPRGHYKLNS---------QKKSTILNTAVPIGSCVSKCHTDKGSISTTKPFQNISRISIGDCPKYVKQGSLKLATGMRNIPEKAT----RGLFGAIAGFIENGWQGLIDGWYGFRHQNAEGTGTAADLKSTQAAIDQINGKLNRLIEKPNEKYHQIEKEFEQVEGRIQDLEKYVEDTKIDLWSYNAELLVALENQHTIDVTDSEMNKLFERVRHQLRENAEDKGNGCFEIFHQCDNSCIESIRNGTYDHDIYRDEAINNRFQIQGVKLIQG--YKDIILWFSFSISCFLLVALLLAFILWACQNGNIRCQICI

>A/Turkey/Italy/220158/2002/H7N3

----------DKICLGHHAVSNGTKVNTLTERGVEVVNATETVERTNVPRICSK-GKRTVDLGQCGLLGTITGPPQCDQFLEF-SADLIIERRE-GSDVCYPGKFVNEEALRQILRESGGIDKETMGF---TYSGIRTN-GATSACRR--SGSSFYAEMKWLLSNTDNAAFPQMTKSYKNTRKDPALIIWGIHHSGSTTEQTKLYGSGNKLITVGSSNYQQSFVPSPGARPQVNGQSGRIDFHWLMLNPNDTVTFSFNGAFIAPDRASFLR----------GKSMGIQSSVQVDANCEGDCYHSGGTIISNLPFQNINSRAVGKCPRYVKQESLMLATGMKNVPEIPKG---RGLFGAIAGFIENGWEGLIDGWYGFRHQNAQGEGTAADYKSTQSAIDQITGKLNRLIEKTNQQFELIDNEFTEVEKQIGNVINWTRDSMTEVWSYNAELLVAMENQHTIDLADSEMNKLYERVKRQLRENAEEDGTGCFEIFHKCDDDCMASIRNNTYDHSRYREEAMQNRIQIDPVKLSSG--YKDVILWFSFGASCFILLAIAMGLVFICVKNGNMRCTICI

>A/Netherlands/219/2003/H7N7

----------DKICLGHHAVSNGTKVNTLTERGVEVVNATETVERTNVPRICSK-GKRTVDLGQCGLLGTITGPPQCDQFLEF-SADLIIERRE-GSDVCYPGKFVNEEALRQILRESGGIDKETMGF---TYSGIRTN-GTTSACRR--SGSSFYAEMKWLLSNTDNAAFPQMTKSYKNTRKDPALIIWGIHHSGSTTEQTKLYGSGNKLITVGSSNYQQSFVPSPGARPQVNGQSGRIDFHWLILNPNDTVTFSFNGAFIAPDRASFLR----------GKSMGIQSEVQVDANCEGDCYHSGGTIISNLPFQNINSRAVGKCPRYVKQESLLLATGMKNVPEIPKRRR-RGLFGAIAGFIENGWEGLIDGWYGFRHQNAQGEGTAADYKSTQSAIDQITGKLNRLIEKTNQQFELIDNEFTEVERQIGNVINWTRDSMTEVWSYNAELLVAMENQHTIDLADSEMNKLYERVKRQLRENAEEDGTGCFEIFHKCDDDCMASIRNNTYDHSKYREEAIQNRIQIDPVKLSSG--YKDVILWFSFGASCFILLAIAMGLVFICVKNGNMRCTICI

>A/mallard/bavaria/3/2006/H10N7

---------LDKICLGHHAVANGTIVKTLTNEQEEVTNATETVESTSLNRLCMK-GRNHKDLGNCHPIGMLIGTPACDLHLTG-TWDTLIEREN-AIAYCYPGATVNEEALRQKIMESGGISKISTGF---TYGSSINSAGTTKACMR-NGGNSFYAELKWLVSKSKGQNFPQTTNTYRNTDTAEHIIMWGIHHPSSTQEKNDLYGTQSLSISVGSSTYQNNFVPVVGARPQVNGQSGRIDFHWTLVQPGDNITFSHNGGLIAPSRVSKLI----------GRGLGIQSDAPIDNNCESKCFWRGGSINTRLPFQNLSPRTVGQCPKYVNKKSLMLATGMRNVPEIMQG---RGLFGAIAGFIENGWEGMVDGWYGFRHQNAQGTGQAADYKSTQAAIDQITGKLNRLIEKTNTEFESIESEFSEIEHQIGNVINWTKDSITDIWTYQAELLVAMENQHTIDMADSEMLNLYERVRKQLRQNAEEDGKGCFEIYHACDDSCMESIRNNTYDHSQYREEALLNRLNINPVTLSSG--YKDIILWFSFGASCFVLLAVVMGLVFFCLKNGNMRCTICI

>A/mallard/Astrakhan/263/1982/H14N5

-QITNGTTGNPIICLGHHAVENGTSVKTLTDNHVEVVSAKELVETNHTDELCPS-PLKLVDGQDCDLINGALGSPGCDRLQDT-TWDVFIERPT-AVDTCYPFDVPDYQSLRSILASSGSLEFIAEQF---TWNGVKVD-GSSSACLR-GGRNSFFSRLNWLTKATN-GNYGPINVTKENTGSYVRLYLWGVHHPSSDNEQTDLYKVATGRVTVSTRSDQISIVPNIGSRPRVRNQSGRISIYWTLVNPGDSIIFNSIGNLIAPRGHYKISK---------STKSTVLKSDKRIGSCTSPCLTDKGSIQSDKPFQNVSRIAIGNCPKYVKQGSLMLATGMRNIPGKQA----RGLFGAIAGFIENGWQGLIDGWYGFRHQNAEGTGTAADLKSTQAAIDQINGKLNRLIEKTNEKYHQIEKEFEQVEGRIQDLEKYVEDTKIDLWSYNAELLVALENQHTIDVTDSEMNKLFERVRRQLRENAEDQGNGCFEIFHQCDNNCIESIRNGTYDHNIYRDEAINNRIKINPVTLTMG--YKDIILWISFSMSCFVFVALILGFVLWACQNGNIRCQICI

>A/duck/Australia/341/1983/H15N8

----------DKICLGHHAVANGTKVNTLTEKGVEVVNATETVEITGINKVCTK-GKKAVDLGSCGILGTIIGPPQCDSHLKF-KADLIIERRN-SSDICYPGKFTNEEALRQIIRESGGIDKEPMGF---RYSGIKTD-GATSACKR--TVSSFYSEMKWLLSSKANQVFPQLNQTYRNNRKEPALIVWGVHHSSSLDEQNKLYGAGNKLITVGSSKYQQSFSPSPGDRPKVNGQAGRIDFHWMLLDPGDTVTFTFNGAFIAPDRATFLRSNAPSGVEYNGKSLGIQSDAQIDESCEGECFYSGGTINSPLPFQNIDSWAVGRCPRYVKQSSLPLALGMKNVPEKIHT---RGLFGAIAGFIENGWEGLIDGWYGFRHQNAQGQGTAADYKSTQAAIDQITGKLNRLIEKTNTQFELIDNEFTEVEQQIGNVINWTRDSLTEIWSYNAELLVAMENQHTIDLADSEMNKLYERVRRQLRENAEEDGTGCFEIFHRCDDQCMESIRNNTYNHTEYRQEALQNRIMINPVKLSGG--YKDVILWFSFGASCVMLLAIAMGLIFMCVKNGNLRCTICI
